# On Acetous Ablution in Typhus and Synochus, &c.

**Published:** 1802-04

**Authors:** Hugh Moises


### *On Acetous Ablution in Typhus and Synochus, &c.*

### To Dr. BRADLEY.

DEAR SIR,

THE internal ufe of vegetable acids was well known to the ancients; and they were held by them as a beverage highly falutary, not only in difeafes where a putrefcent and diffolved ftate of the blood and juices was fuppofed to exift, but alfo as a common drink in health, to guard againft the acceffion of fuch difeafes. Thus we find the Roman labourers and foldiers had a daily allowance of meagre wine, or vinegar and water, called *pofca* or *lora*. (See Varr. Cato, Pliny, and others.)

The exhibition of vegetable acids was equally known and adopted by the phyficians of fubfequent ages, and has been continued to the prefent day in many difeafes which fhew a tendency to putrefaction, as in the fcurvy, &c. That however produced by fermentation, feems better adapted to medical purpofes than the native acids, for it is known to arreft or pre-

 vent vent the procefs of fermentation, and is admirably calculated to remove that alkaline ftate of the fluids which is found to exift in fome perfons even in health, and in many cafes of difeafe. The *acetum vini* is the moft powerful of thofe produced by fermentation, and that on which I am now to deliver my fentiments. When taken into the ftomach in moderate quantities it is well known to promote digeftion, and even to prevent thofe flatulencies which might otherwife arife from the decompofition of the vegetable part of our diet. It is equally certain that when taken to a given extent it promotes the falutary excretions, and when in greater quantity becomes a powerful refrigerant. Upon a due confideration of thefe well known effects, together with what had been produced by the affufion of water in febrile difeafes, I was led to fubftitute the *acetum vini* as poffeffing an advantage over water by reafon of its greater antifeptic powers, and to prefer frequent ablution to affufion. This treatment I have directed in many inftances of typhus, fynochus, &c. both in military and private practice fince the fpring of 1794, with the moft marked advantage, and in which a happy refult not only confirms the beneficial influence of this acid, beyond any other medicine with which I am acquainted, but has led me to confider it as meriting the general attention of the profeffion, not only in thefe difeafes but in all others which by their analogy may afford the fame obvious indications of cure. That your readers may judge for themfelves, I have felected two cafes, which are recent. In the firft, it will be feen that the ablution produced effects equally rapid and beneficial, nor could the quantity of bark thrown into the inteftinum rectum be fuppofed to produce much effect in fo fhort a time. The cafe, however, will fpeak for itfelf.

Sarah Taylor, aged about twenty months, was feized with fymptoms of pyrexia on the 25th of October laft. Having enjoyed good general health from her birth, and not having been expofed to the atmofphere of any perfon labouring under contagious fever, her father (a refpectable furgeon and apothecary) gave her a gentle aperient, and diluent febrifuge medicines. The fymptoms however gained ground, and on the 27th, excited confiderable alarm. I was requefted to fee the child, whom I found with every fymptom of exquifitely formed typhus, all of which were heightened by fuppreffion of the natural excretions. I prefcribed $\frac{1}{16}$ of a grain of antim. tartarizat. to be given every hour until an evacuation fhould be procured, after which an injection of extract. cinchonæ Americanæ ʒj. with decoct. cinchonæ ℥iv. was thrown up and repeated every fixth hour. On the following day I faw her again, when vibices were found to have occupied the anterior, pofterior, and exterior exterior furface of each foot, and extending a confiderable way up the leg; they had affumed the livid hue and the extremities were cold, and the cuticle had begun to feparate on the fuperior part of the great toe. We agreed in opinion that little or no hope was to be entertained of her furviving many hours. The anxiety of the parent was extreme; he confidered every effort we might make to fave the child abortive, and only tending to harrafs the little fufferer. My hopes were but little more flattering; yet I preffed the continuance of the injections, and that the child fhould be fubmitted to *frequent and copious ablution with vinegar*. My advice was complied with by the father, rather I believe through compliment to me and a wifh to leave nothing undone, than from any hope he entertained of its even mitigating the fufferings of the child; and the mother, a woman of great good fenfe and fine feelings, catching a ray of hope from the propofed plan, performed the ablutions herfelf, frequent and copious, throughout the night. The following day furprized us with evident amendment by the difappearance of the vibices. The ablutions were continued without any other collateral aid, but that of wine and nutritive diet; her return to health was effected in a few days, and her convalefcence has remained complete to the prefent hour. In tracing out the caufe of the difeafe, we could fix upon none other than infection or contagion from *fomites*, the father having, two or three days before the fickening of the child, returned from vifiting a patient fome miles off who then laboured under fcarlatina anginofa, and without taking off his Spencer, began nurfing the child. If this were the cafe, why had not this child the characteriftic appearances of fcarlatina? Does not this cafe feem to prove that contagion from one difeafe is capable of producing a difeafe different from, and wanting the pathognomonic figns of that from whence it derived its fource? The reft of the family efcaped infection.

I. King, jun. 23 years of age, by occupation a farmer, had laboured under rheumatifmus acutus early in laft autumn, from which he had been relieved. On the 25th of December following I was called in; he had been confined for about fifteen days under the care of Mr. Brown, an apothecary in the neighbourhood, on whofe judgement of the cafe I could place the moft unlimited reliance. The fymptoms from the commencement had been thofe of *typhus gravior*. He had given calomel conjoined with the antimonial powder, and the bark liberally. The difeafe however had proceeded without remiffion, though by gradual progreffion, till the day on which I was called in. I found him in a ftate which admitted but a very cautious prognofis. The bowels were not free, although he had taken the

 calomel calomel and antimonial powder daily; his pulfe 108, and feeble in the extreme; much head-ach, and occafional flight delirium. The tongue and teeth covered with a thick, dry, and black fordes; the fauces and tonfils much inflamed, and deglutition very difficult; urine pale, and fmall in quantity; cold colliquative fweats, and great proftration of ftrength. I directed a bolus to be taken every night, confifting of pil. faponis gr. viij. calomelas gr. j ß. opii gr. ß. The cortex was continued as before or with but very little alteration, and he took Port wine with the citric acid, ad libitum. But my principal fupport was placed on the acetous ablution, which was repeated every third hour, fo as to confume from four to fix pints of vinegar in the courfe of the day and night. I faw him again on the 27th, when the pulfe was at 80, and fuller withal. Bowels regular; tongue more clean and moift; inflammations of the tonfils, &c. fubfiding; urine natural; no head-ach or delirium. A moift and natural perfpiration had fucceeded the fecond ablution, and he felt in every refpect better. The whole plan was directed to be continued. On the 29th I found him advancing rapidly to a ftate of convalefcence. The bolus was directed to be continued without the calomel, and the reft of the treatment as before. On the 1ft of January I took my leave, defiring him to continue his bark and ablutions for a few days, taking his nutriment at frequent periods, but with caution as to the quantities he might take at one time. On the 18th he came to my houfe, a diftance of feven miles, in perfect health, except a little weaknefs of the lower extremities. I faw him two days ago, and learnt from him that he had, fince the 18th, purfued his accuftomed avocations in the farm, and was in perfect health. The foothing effect of the ablution, applied cold, was fuch as will ever leave a lafting impreffion on his memory; and although much aid was derived from the cinchona, wine, and acid taken internally, I do not believe he would have recovered without the ablution.

It may be expected I fhould give fome opinion as to the modus operandi of acetum in thefe cafes. I am inclined to believe that its primary action is that of a ftimulant, but in fuch degree only as may excite an healthy action of the folids, efpecially of the inhalents and abforbents. I believe in this way it is readily taken into the habit, and promotes the action of the whole vafcular fyftem. That its powers are antifeptic and aftringent there can fcarcely be any fhadow of doubt; and that it may form a *tertium quid*, deftructive of contagion, I am led to infer from repeatedly, I might fay uniformly obferving, that, cæteris paribus, where it had been liberally ufed, the influence of the contagion has appeared to be very limited, or altogether deftroyed deſtroyed, inſomuch that actual contact would not produce the diſeaſe, where the adminiſtration of vinegar had been ample to the patient, his apartment, and every thing about him. I ſhall, in a few weeks, ſubmit to you ſome Obſervations on the efficacy of Elaterium and Digitalis in certain diſeaſes. In the interim, I am, &c.

*9th March*, 1802. HUGH MOISES, M. D.

